# A cfDNA fragmentomics classifier for noninvasive differentiation of benign and malignant renal masses

**DOI:** 10.1186/s43556-026-00568-4

**Published:** 2026-09-07

**Authors:** Linfei Li, Cong Wang, Song Wang, Jun Zheng, Jian Fu, Ling Wei, Juan Shen, Yuwei Li, Xinyue Hong, Chunman Wu, Wei Chen, Wei Gong, Yongquan Wang

**Affiliations:** 1 Department of Urology, The First Affiliated Hospital of Army Medical University, Chongqing, 400038 China; 2 Geneseeq Research Institute, Nanjing Geneseeq Technology Inc., Nanjing, 210032 China; 3 Department of Urology of Jiangbei Campus, The First Affiliated Hospital of Army Medical University (The 958th Hospital of Chinese People’s Liberation Army), Chongqing, 400020 China; 4 Department of Biochemistry and Molecular Biology, College of Basic Medical Sciences, Army Medical University, Chongqing, 400038 China

**Keywords:** Renal tumor, Cell-free DNA, Fragmentomics, Tumor classification, Liquid biopsy

## Abstract

Noninvasive differentiation of malignant and benign renal masses remains a major clinical challenge, particularly for radiologically indeterminate lesions. Here, we developed and validated a plasma cell-free DNA (cfDNA) fragmentomics-based machine learning classifier for renal mass characterization. The model was trained on 331 participants (171 cancer, 160 benign) and independently validated on 144 participants (73 cancer, 71 benign). Three cfDNA fragmentation features, including copy number variation (CNV), fragmentation-based methylation (FRAGMA), and nucleosome footprint (NF), derived from low-pass whole-genome sequencing, were integrated into an ensemble framework. The model achieved strong discriminative performance, with area under the curve (AUC) values of 0.956 in the training cohort and 0.946 in the validation cohort, outperforming individual feature-based models. At a predefined operating threshold corresponding to 90% sensitivity, specificity reached 0.90 and 0.87, respectively. Notably, most cancer samples exhibited low tumor fraction (TF < 3%), yet the model maintained robust performance in low-TF samples (AUCs: 0.952 and 0.941, respectively). Performance remained consistent across tumor stage, grade, and histological subtypes. The classifier also demonstrated potential clinical utility in diagnostically challenging settings, including lipid-poor angiomyolipoma and oncocytoma, with 12 of 13 oncocytoma samples correctly classified in an independent cohort. In addition, the model correctly identified 85.3% of benign masses > 4 cm, for which surgical intervention is more commonly considered, and 84.6% of malignant tumors ≤ 4 cm, for which management can be challenging. Collectively, these findings support cfDNA fragmentomics as a promising noninvasive liquid biopsy approach for renal mass evaluation and clinical decision-making.

**Supplementary Information:**

The online version contains supplementary material available at https://doi.org/10.1186/s43556-026-00568-4.

## Introduction

Renal masses comprise a diverse spectrum of lesions, ranging from benign entities such as cysts, oncocytomas, and angiomyolipomas (AML) to malignant tumors such as renal cell carcinoma (RCC) [1]. Accurate discrimination between benign and malignant renal masses is of paramount clinical importance. Over the past two decades, the increasing use of abdominal imaging has led to a substantial rise in the incidental detection of renal masses, a considerable proportion of which are ultimately found to be benign after surgical resection [2, 3]. However, the inability to reliably determine malignancy preoperatively often results in overtreatment. Unnecessary surgical intervention for benign lesions exposes patients to avoidable perioperative morbidity and potential long-term impairment of renal function [3–5]. Conversely, underdiagnosis of malignant lesions may delay curative intervention and worsen oncologic outcomes.

Despite advances in imaging modalities, preoperative characterization of renal masses remains challenging. Conventional imaging techniques such as contrast-enhanced computed tomography (CT) and magnetic resonance imaging (MRI) provide valuable anatomical and perfusion-based information, yet the radiological overlap between benign oncocytoma and chromophobe RCC (chRCC), or between lipid-poor AML and clear cell RCC (ccRCC), frequently results in diagnostic ambiguity [6–10]. Although percutaneous renal mass biopsy can provide confirmation, it is invasive and may be limited by sampling bias and intratumoral heterogeneity [1]. Consequently, many patients undergo surgery without a definitive preoperative diagnosis. These limitations underscore the urgent clinical need for a noninvasive and accurate biomarker to distinguish benign from malignant renal masses and guide individualized treatment strategies.

Circulating tumor DNA (ctDNA), the tumor-derived fraction of cell-free DNA (cfDNA), is widely used for mutation- and methylation-based liquid biopsy. However, ctDNA abundance can be extremely low in early-stage or small-volume tumors, limiting the sensitivity of mutation-centric assays. Fragmentomic analysis examines cfDNA fragment sizes, end motifs, and genomic positioning to capture chromatin- and nucleosome-related signals enriched in tumor-derived fragments [11–15]. Fragmentomic signatures have successfully aided early cancer detection and tissue-of-origin classification across multiple tumor types [16–19]. In the context of renal masses, cfDNA-based fragmentomics provides an opportunity to enhance tumor-specific signals, enabling noninvasive discrimination between benign and malignant lesions and supporting the development of predictive models complementary to imaging and biopsy.

Building on these insights, we hypothesized that cfDNA fragmentomic features, even in the context of low tumor fraction (TF), could capture tumor-associated biological signals sufficient for distinguishing benign from malignant renal masses. To test this hypothesis, we developed a machine learning-based cfDNA fragmentomics framework and evaluated it in independent cohorts of patients with renal masses. This framework integrates multiple fragmentomic features to improve robustness across clinically relevant subgroups, including early-stage tumors, low tumor burden cases, and histologically ambiguous lesions such as oncocytoma, which are particularly challenging to differentiate using conventional imaging. The model demonstrated strong discriminative performance and clinically meaningful stratification of renal masses. These findings suggest that cfDNA fragmentomics may help shift the current diagnostic paradigm from morphology-based assessment toward a molecularly informed, minimally invasive approach, with potential to improve preoperative risk stratification and reduce unnecessary surgical interventions.

## Results

### Study design and participant characteristics

**Fig. 1:**
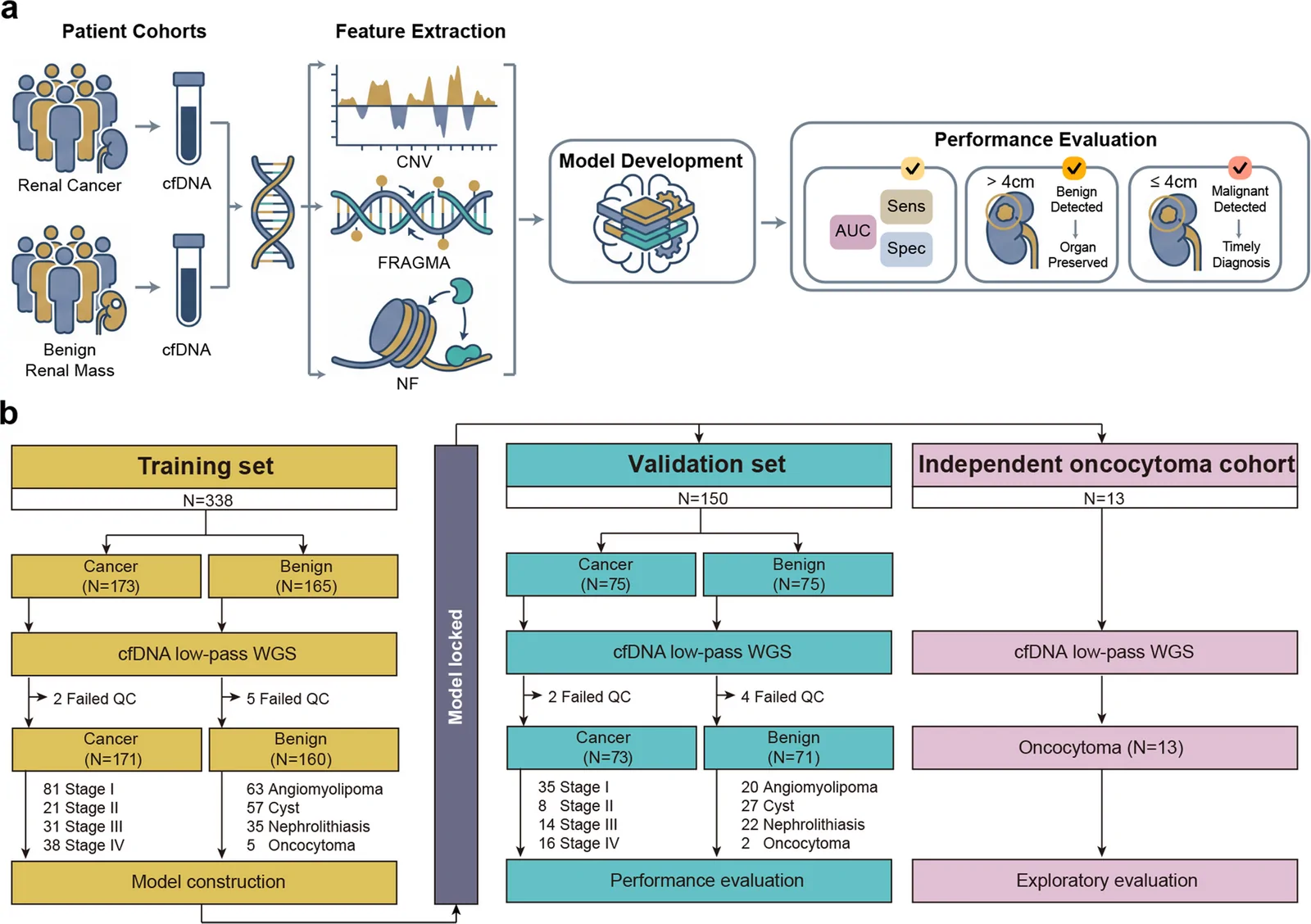
Overview of the study design. **a** Schematic representation of the study workflow. Cell-free DNA (cfDNA) was isolated from blood samples of patients with renal cancer or benign renal masses. Fragmentomic features, including copy number variation (CNV), Fragmentation-based methylation (FRAGMA), and nucleosome footprint (NF), were derived from low-pass whole-genome sequencing (WGS). These features were integrated into a machine-learning framework to distinguish malignant from benign conditions. Model performance was evaluated using metrics such as area under the curve (AUC), sensitivity, and specificity, with particular emphasis on clinically relevant size-defined subgroups (> 4 cm and ≤ 4 cm). **b** Flowchart of patient enrollment and cohort construction. Following sequencing quality control, a training cohort (N = 331) and an independent validation cohort (N = 144) were included for model development and performance evaluation, respectively. An additional independent cohort of 13 patients with pathologically confirmed oncocytoma was analyzed separately for exploratory evaluation of model performance in this underrepresented benign renal tumor subtype

We aimed to develop and validate a machine learning model that noninvasively differentiates malignant from benign renal masses using fragmentomic features derived from plasma cfDNA. The initial training cohort comprised 173 participants with renal cancer and 165 individuals with benign renal masses, all of whom were pathologically diagnosed after surgery (Fig. 1). An independent evaluation cohort of 150 participants, 75 with renal cancer and 75 with benign masses, was recruited at the same hospital to assess model performance. Following quality control, 331 participants (171 cancer, 160 benign) in the training set and 144 participants (73 cancer, 71 benign) in the evaluation set were retained for analysis.

Baseline characteristics of the participants are summarized in Table 1. In the training cohort, the median age was 55 years for both cancer and benign groups. Cancer patients were predominantly male, with an approximate 2:1 male-to-female ratio, a pattern consistent with previously reported epidemiologic data for RCC [20]. Most participants were normotensive and had no history of smoking. Among cancer patients, nearly 60% presented with stage I/II disease, and 46.8% had renal masses ≤ 4 cm. Pathologically, 75.4% of malignant masses were classified as clear cell renal cell carcinoma (ccRCC), while each of the remaining subtypes, including chromophobe RCC (chRCC), papillary RCC (pRCC), and several less common entities (e.g., *TFE3*-rearranged RCC, eosinophilic solid and cystic RCC, and acquired cystic disease-associated RCC) that were grouped as “others” in this study, individually accounted for less than 10%. The distribution of radiological tumor sizes in benign masses was similar, with AML as the most common subtype (39.4%), followed by cysts (35.6%), nephroliths (21.9%), and oncocytomas (3.1%). Statistical comparisons of baseline characteristics between the training and validation cohorts revealed no significant differences in the measured characteristics for either the cancer or benign groups.

**Table 1:** Baseline characteristics of participants

	**Training cohort (N = 331)**		**Validation cohort (N = 144)**		**Training vs. Validation**	
	**Cancer**	**Benign**	**Cancer**	**Benign**	**Cancer**	**Benign**
**Total, n (%)**	171 (51.70%)	160 (48.30%)	73 (50.69%)	71 (49.31%)		
**Age, median [range]**	55 [25,78]	55 [30,80]	50 [25,75]	51 [30,70]	0.296	0.280
**Sex**					0.971	0.330
Female	63 (36.8%)	96 (60.0%)	26 (35.6%)	37 (52.1%)		
Male	108 (63.2%)	64 (40.0%)	47 (64.4%)	34 (47.9%)		
**Hypertension status**					0.157	0.178
Grade 1	12 (7.0%)	2 (1.3%)	12 (16.4%)	3 (4.2%)		
Grade 2	20 (11.7%)	4 (2.5%)	7 (9.6%)	3 (4.2%)		
Grade 3	17 (9.9%)	10 (6.3%)	6 (8.2%)	1 (1.4%)		
Normotensive	122 (71.3%)	144 (90.0%)	48 (65.8%)	64 (90.1%)		
**BMI**					0.466	0.842
< 25.0 kg/m^2^	96 (56.1%)	101 (63.1%)	35 (47.9%)	43 (60.6%)		
≥ 25.0 kg/m^2^	72 (42.1%)	55 (34.4%)	37 (50.7%)	26 (36.6%)		
Unknown	3 (1.8%)	4 (2.5%)	1 (1.4%)	2 (2.8%)		
**Triglycerides**					0.178	0.823
< 1.7 mmol/L	97 (56.7%)	97 (60.6%)	31 (42.5%)	40 (56.3%)		
≥ 1.7 mmol/L	55 (32.2%)	54 (33.8%)	28 (38.4%)	25 (35.2%)		
Unknown	19 (11.1%)	9 (5.6%)	14 (19.2%)	6 (8.5%)		
**Total cholesterol**					0.146	0.807
< 5.2 mmol/L	90 (52.6%)	88 (55.0%)	42 (57.5%)	36 (50.7%)		
≥ 5.2 mmol/L	62 (36.3%)	63 (39.4%)	17 (23.3%)	29 (40.9%)		
Unknown	19 (11.1%)	9 (5.6%)	14 (19.2%)	6 (8.4%)		
**Smoking history**					0.641	> 0.999
Never	117 (68.4%)	121 (75.6%)	47 (64.4%)	53 (74.6%)		
Current/former	54 (31.6%)	39 (24.4%)	26 (35.6%)	18 (25.4%)		
**TNM stage**					0.990	~
I	81 (47.4%)		35 (47.9%)			
II	21 (12.3%)		8 (11.0%)			
III	31 (18.1%)		14 (19.2%)			
IV	38 (22.2%)		16 (21.9%)			
**WHO/ISUP grade**					0.766	~
I	23 (13.5%)		7 (9.6%)			
II	49 (28.7%)		22 (30.1%)			
III	45 (26.3%)		24 (32.9%)			
IV	19 (11.1%)		6 (8.2%)			
Unknown	5 (2.9%)		1 (1.4%)			
Not applicable	30 (17.5%)		13 (17.8%)			
**Radiological size**					0.110	0.472
Size ≤ 4 cm	80 (46.8%)	79 (49.4%)	24 (32.9%)	36 (50.7%)		
4 cm < Size ≤ 10 cm	76 (44.4%)	67 (41.9%)	39 (53.4%)	32 (45.1%)		
Size > 10 cm	15 (8.8%)	14 (8.8%)	10 (13.7%)	3 (4.2%)		
**Pathological type**					0.172	~
ccRCC	129 (75.4%)		50 (68.5%)			
chRCC	16 (9.4%)		10 (13.70%)			
pRCC	12 (7.0%)		10 (13.70%)			
Others	14 (8.2%)		3 (4.1%)			
**Benign type**					~	0.325
Angiomyolipoma		63 (39.4%)		20 (28.2%)		
Cyst		57 (35.6%)		27 (38.0%)		
Nephrolithiasis		35 (21.9%)		22 (31.0%)		
Oncocytoma		5 (3.1%)		2 (2.8%)		

*Abbreviations*: *BMI* body mass index, *TNM* tumor, node, metastasis, *WHO* World Health Organization, *ISUP* International Society of Urological Pathology, *ccRCC* clear cell renal cell carcinoma, *chRCC* chromophobe renal cell carcinoma, *pRCC* papillary renal cell carcinoma

### cfDNA fragmentation features

**Fig. 2:**
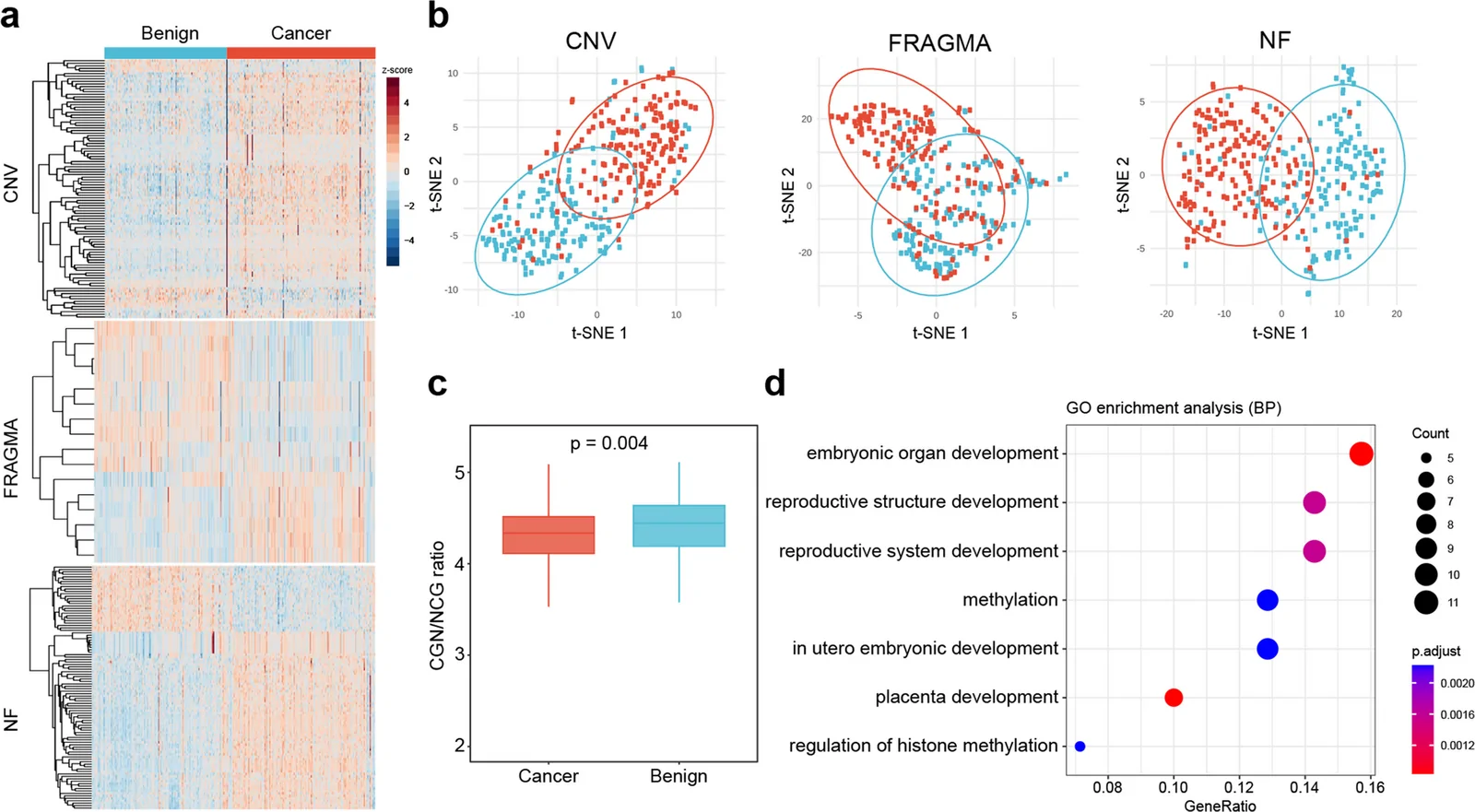
cfDNA fragmentation profiles. **a** Heatmap of normalized cfDNA feature values (z-scores) for copy number variation (CNV), fragmentation-based methylation (FRAGMA), and nucleosome footprint (NF) in participants with malignant or benign renal masses. **b** t-distributed stochastic neighbor embedding (t-SNE) visualization of CNV, FRAGMA, and NF features, illustrating the separation between cancer and benign samples. **c** Box plot comparing inferred methylation levels between cancer and benign masses. **d** Gene Ontology (GO) enrichment analysis of genes associated with transcription factors exhibiting cancer-related nucleosome footprint patterns, showing the top enriched biological processes (BP)

Three cfDNA fragmentation features, specifically copy number variation (CNV), fragmentation-based methylation (FRAGMA), and nucleosome footprint (NF), were derived from low-pass WGS data and used to construct models distinguishing malignant from benign renal masses. When evaluated individually, all three features demonstrated discriminatory power between cancer and non-cancer patients (Fig. 2a, b). Analysis of the FRAGMA feature revealed that cancer patients exhibited significantly lower inferred methylation compared to those with benign masses (Fig. 2c), consistent with global hypomethylation as a hallmark of cancer. Functional enrichment analysis of genes associated with transcription factors exhibiting cancer-related NF patterns showed enrichment in biological processes related to embryonic organ development, reproductive system and structure development, and methylation-related processes (Fig. 2d).

We next evaluated whether integrating these feature dimensions into an ensemble model could further optimize diagnostic performance. The resulting full model achieved AUCs that outperformed both the single-feature models and all “drop-one” configurations (Fig. S1a). Notably, the permutation importance analysis provided additional support for the inclusion of all three feature dimensions (Fig. S1b). By systematically permuting each feature group while preserving the others, we demonstrated that the model’s optimal performance in the validation cohort depends on the unique predictive information contributed by each modality. Permutation of FRAGMA resulted in the largest performance reduction, identifying it as the primary predictive driver. Importantly, permutation of CNV and NF also consistently reduced model performance relative to the original validation AUC, with all permutation-derived AUCs remaining below the unpermuted performance, supporting their non-redundant and complementary predictive value.

Collectively, these findings suggest that cfDNA fragmentation patterns reflect not only tumor biology but also broader epigenetic and developmental programs associated with malignancy, underscoring their potential for noninvasive cancer detection. These results further support the integration of multiple fragmentomic dimensions and the use of a holistic multi-feature framework to improve diagnostic accuracy and model robustness.

### Model performance and operating point selection

**Fig. 3:**
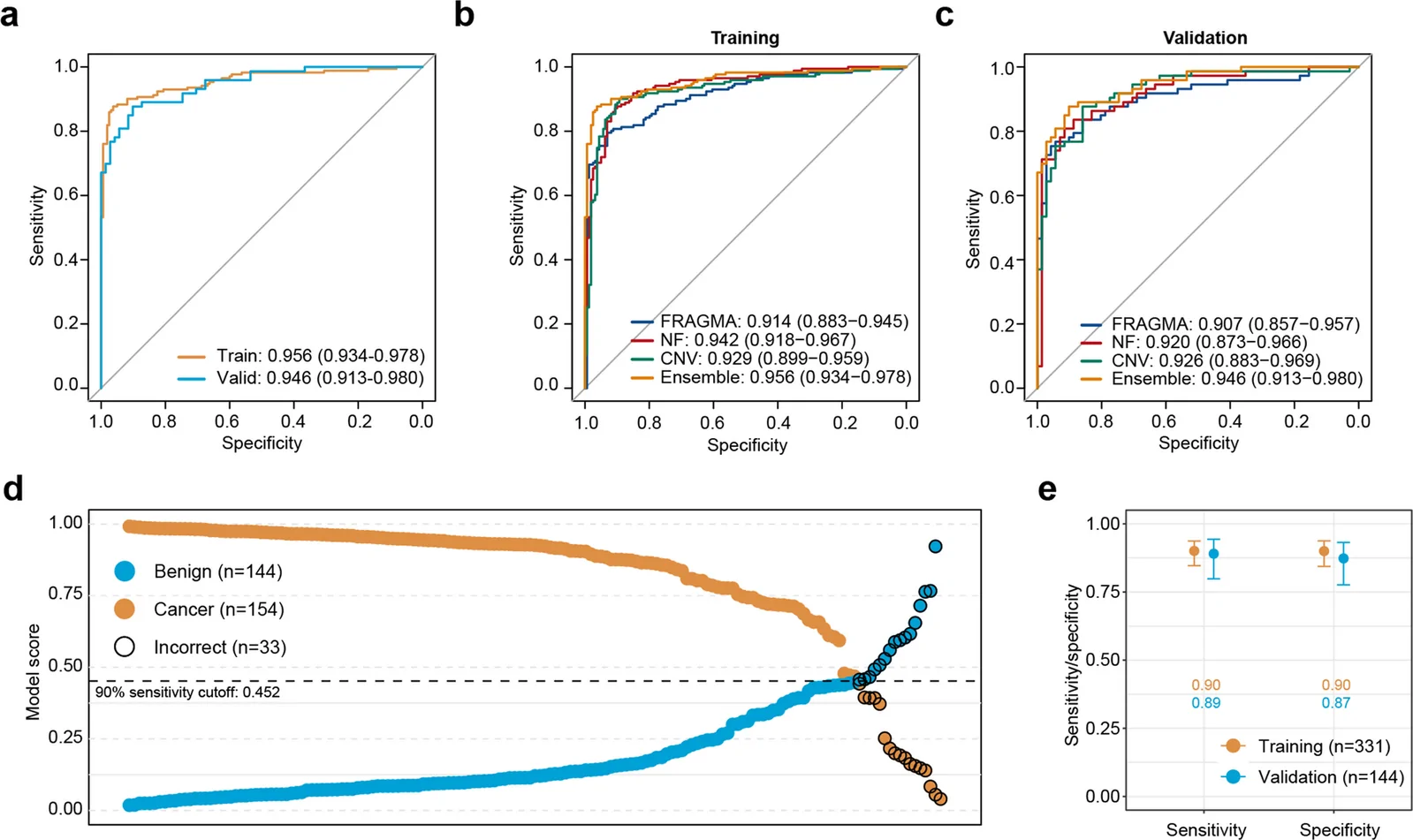
Model performance evaluation. **a** Receiver operating characteristic (ROC) curves for the predictive model in the training and validation cohorts. **b**, **c** ROC curves for the ensemble model versus single-feature models in the training (**b**) and validation (**c**) cohorts. **d** Prediction scores for individuals in the training cohort. The cutoff value was optimized to achieve 90% sensitivity. Scores are presented in ascending order for participants with benign renal masses and descending order for renal cancer patients. Incorrect predictions are marked with a black outline on the right. **e** Sensitivity and specificity of the predictive model across datasets

The final model integrating all three cfDNA features demonstrated robust predictive performance across datasets. In the training cohort, the model achieved an AUC of 0.956 (95% CI: 0.934–0.978), while in the validation cohort, the AUC was 0.946 (95% CI: 0.913–0.980) (Fig. 3a). Notably, the ensemble model consistently outperformed all individual feature-based models in both cohorts (Fig. 3b, c). Decision curve analysis (DCA) demonstrated that the ensemble model yielded higher standardized net benefit than the “treat-all”, “treat-none”, and single-feature strategies across most threshold probabilities in both datasets, supporting its potential clinical utility for decision-making (Fig. S2a, b).

To define a clinically meaningful operating point, we performed sensitivity escalation analysis and observed a clear trade-off between sensitivity and specificity (Table S1). Increasing sensitivity to higher levels further reduced false-negative tumors but resulted in substantial losses in specificity, while the additionally detected tumors were not preferentially characterized by adverse clinicopathological features (Table S2). Based on this trade-off, a cutoff corresponding to 90% sensitivity was selected as the primary operating point (cutoff = 0.452), providing a balanced trade-off between sensitivity and specificity. At this threshold, the model achieved specificities of 0.90 in the training cohort and 0.87 in the validation cohort (Fig. 3d, e).

To further assess clinical utility, we evaluated predictive performance across a range of malignancy prevalence settings (Table S3). In higher-prevalence scenarios (70–80%), representative of clinically enriched populations, the model demonstrated strong positive predictive value (PPV; 0.94–0.97) and moderate negative predictive value (NPV; 0.67–0.77) in the validation cohort, supporting its potential utility in preoperative risk stratification and improving diagnostic confidence in high-risk patients. In contrast, under a low-prevalence scenario (5%), the model maintained an excellent NPV of 0.99, highlighting its potential value as a rule-out tool for identifying patients at a very low likelihood of malignancy and potentially reducing unnecessary longitudinal imaging or invasive biopsy procedures.

### Model robustness across demographics and clinicopathological subgroups

Given the sex imbalance between malignant and benign cases in our cohorts, we first evaluated whether model performance was confounded by sex-related differences. Stratified analyses showed no significant differences in model scores between male and female participants in either cohort (all p > 0.05) (Fig. S3a, b), suggesting comparable model predictions between sexes. In addition, we examined the relationship between the model score and predicted probability of malignancy using logistic regression. The fitted probability curves for males and females exhibited nearly identical slopes in both cohorts (Fig. S3c). The interaction p-values between the model score and sex were non-significant in both the training (p = 0.676) and validation (p = 0.923) cohorts, indicating that the predictive effect of the model score was consistent across sexes. To further validate these findings, we fitted multivariable logistic regression models adjusted for age and sex. The model score remained a strong independent predictor of malignancy, with an adjusted odds ratio (OR) of 21.50 per standard deviation (SD) increase in the training cohort and 15.71 per SD increase in the validation cohort (Fig. S3d). Notably, no significant interaction between model score and sex was observed. Furthermore, ROC analyses stratified by sex demonstrated consistent discriminative power, with highly comparable AUCs between males and females in both the training cohort (0.958 vs. 0.957) and validation cohort (0.950 vs. 0.942) (Fig. S3e). Using the predefined threshold, sensitivity and specificity also remained stable between male and female subgroups (Fig. S3f). We further evaluated whether the model score remained independently associated with malignancy after adjustment for relevant demographic and clinical covariates. The model score remained strongly associated with malignancy in both cohorts in the primary model and after additional adjustment for triglycerides and total cholesterol in the sensitivity model (Fig. S4), supporting the robustness of this association to broader covariate adjustment.

**Fig. 4:**
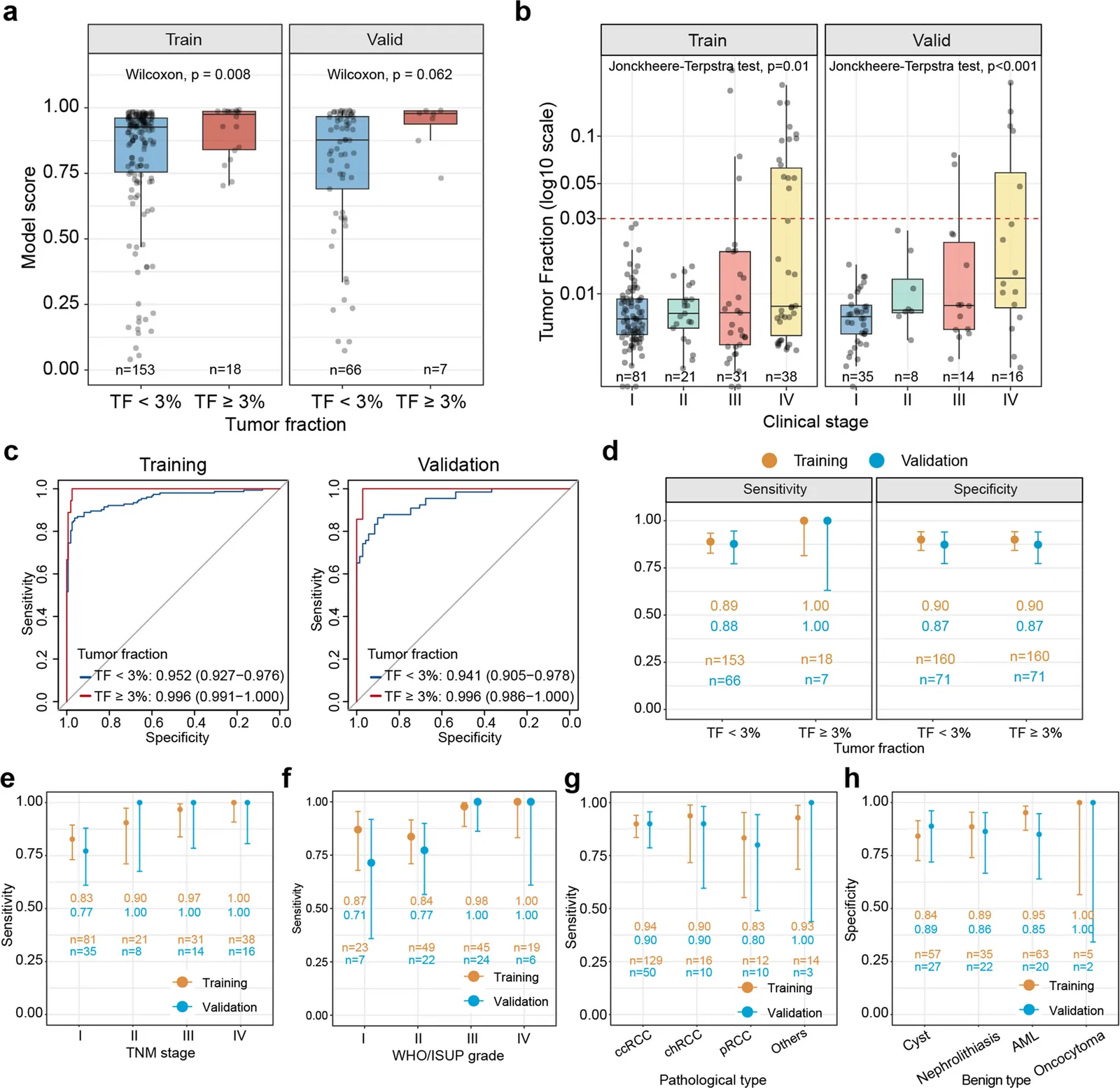
Model robustness across tumor fraction strata and clinicopathological subgroups. **a** Box plots comparing model scores between low tumor fraction (TF < 3%) and high tumor fraction (TF ≥ 3%) subgroups in the training and validation cohorts. **b** Distribution of estimated TF across clinical stages. The red dashed line indicates the 3% TF threshold, with p-values calculated using the two-sided Jonckheere-Terpstra test to assess the trend across stages. **c** Receiver operating characteristic curves stratified by TF levels (< 3% vs. ≥ 3%) in both cohorts. **d** Sensitivity and specificity of the model stratified by TF subgroups. Estimates are based on a predefined cutoff corresponding to 90% sensitivity in the training cohort. **e–h** Sensitivity or specificity of the predictive model in subgroups stratified by TNM stage (**e**), WHO/ISUP grade (**f**), pathological type (**g**), and benign type (**h**)

We next evaluated model performance across the spectrum of tumor burden, given that renal cell carcinoma is generally characterized by low ctDNA shedding. Consistent with this, most cancer samples (89.5% in training and 90.4% in validation) presented with an estimated TF below 3% (Fig. S5a, b). Model scores were significantly higher in the TF ≥ 3% subgroup in the training cohort (Wilcoxon test, p = 0.008), with a similar but non-significant trend observed in the validation cohort (p = 0.062), potentially due to the smaller sample size in the high-TF group (Fig. 4a). To contextualize these TF levels clinically, we examined their distribution across tumor stages and observed a significant increase in TF with advancing disease stage from I to IV (Fig. 4b), although even stage III-IV cases frequently remained within the low-TF range. Despite the challenge, the model maintained strong discriminative power, yielding AUCs of 0.952 and 0.941 for the TF < 3% subgroup in the training and validation cohorts, respectively (Fig. 4c). Using the predefined cutoff, performance remained robust in the low-TF subgroup (sensitivity: 0.88–0.89; specificity: 0.87–0.90), while sensitivity reached 1.00 for samples with TF ≥ 3% in both cohorts (Fig. 4d). Consistently, both model scores and model performance generally increased with advancing TNM stage and WHO/ISUP grade, with lower sensitivity observed in stage I disease compared to later stages (Fig. 4e-h; Fig. S6-S8). Across histopathological subtypes and benign lesion categories, performance remained generally robust but exhibited expected variability among subgroups, reflecting differences in tumor biology and lesion heterogeneity. Collectively, these findings indicate consistent model performance across demographic strata, tumor burden levels, and clinicopathological subgroups.

### Clinical applications of the renal mass classifier

**Fig. 5:**
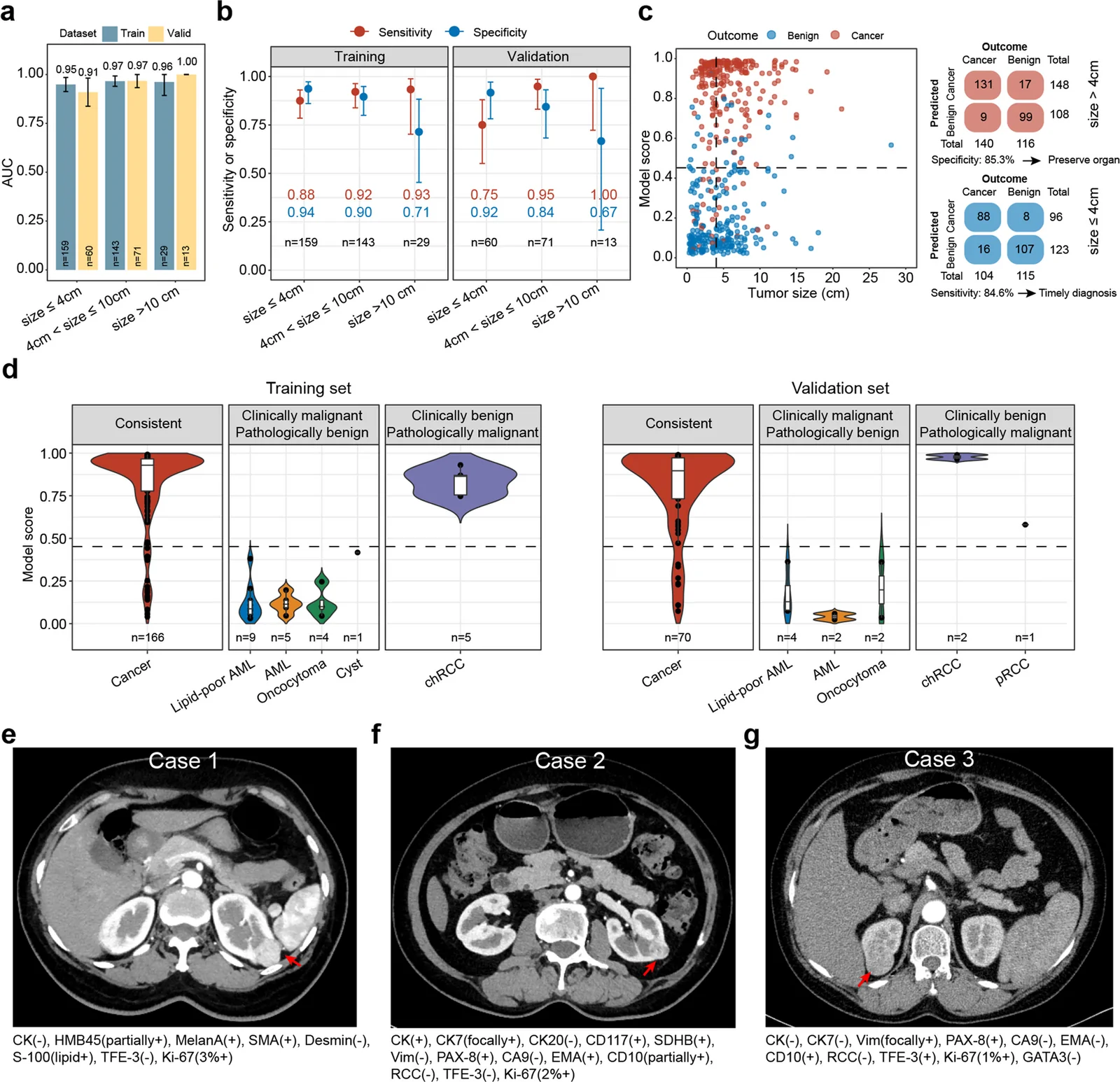
cfDNA-based renal mass classifier for guiding clinical management. **a** Area under the curve (AUC) of the cfDNA-based model across radiological size groups in the training and validation cohorts. **b** Sensitivity and specificity of the model within each tumor size subgroup. **c** Model predictions stratified by tumor size (> 4 cm versus ≤ 4 cm) using the predefined cutoff, showing correct classification of malignant and benign cases. **d** Distribution of model prediction scores for lesions that were consistent versus inconsistent between imaging and pathology across datasets. **e**, **f** Representative imaging and immunohistochemistry (IHC) results from lipid-poor angiomyolipoma (**e**) and oncocytoma (**f**) cases that exhibited radiological features mimicking malignancy. **g** Imaging and IHC results of a patient initially clinically diagnosed with AML but pathologically confirmed as clear cell renal cell carcinoma

We next evaluated the potential clinical utility of the cfDNA fragmentation-based model in the management of patients with renal masses. Tumor size is an important factor in the clinical management of renal masses, although treatment decisions also depend on tumor location, patient characteristics, renal function, and anatomical complexity [1]. In this clinical context, our model achieved AUCs ranging from 0.91 to 1.00 across radiological size subgroups (Fig. 5a). Using the predefined cutoff, the minimum specificity observed across the training and validation sets was 0.92 for masses ≤ 4 cm, 0.84 for masses 4–10 cm, and 0.67 for masses > 10 cm (Fig. 5b). The relatively lower specificity observed in larger tumors should be interpreted with caution, given the limited number of cases in the > 10 cm subgroup and the resulting instability of specificity estimates. Further studies with larger numbers of large benign renal masses are needed to better characterize model performance in this subgroup.

Stratification by the 4 cm threshold further demonstrated the model’s performance in clinically relevant size-defined subgroups (Fig. 5c; Fig. S9). Within the > 4 cm group, in which surgical intervention is more commonly considered in clinical practice, the model correctly identified 85.3% of benign cases (99/116), suggesting potential value in flagging benign lesions that might otherwise be treated surgically. Conversely, among lesions ≤ 4 cm, where surgical decision-making often requires greater caution to balance early cancer detection against potential overtreatment, the model detected 84.6% of malignant cases (88/104), supporting its ability to identify malignant small renal masses in a clinically nuanced subgroup. Performance under higher sensitivity operating points was also evaluated. Both 95% and 98% sensitivity thresholds resulted in a marked reduction in specificity across tumor size subgroups (Fig. S10a, b). Importantly, in the > 4 cm subgroup, higher sensitivity thresholds did not provide a clear advantage, as modest gains in sensitivity were offset by substantially increased false-positive rates among benign lesions (Fig. S10c, d).

Furthermore, 35 cases showed discrepancies between imaging-based and pathological diagnoses, including 27 benign masses and 8 cancers (Fig. 5d). Among the benign lesions, AMLs, particularly lipid-poor AMLs, and oncocytomas were the most common entities mimicking malignancy on imaging, accounting for the majority of imaging-discordant benign cases (Fig. 5e-g). For example, case 1 presented with a solid renal mass that appeared highly suspicious for carcinoma radiologically, yet the final pathological diagnosis confirmed a lipid-poor AML. Case 2 similarly exhibited imaging characteristics suggestive of malignancy, but pathology ultimately identified the lesion as an oncocytoma. In contrast, case 3 appeared benign on imaging and was initially suspected to be AML, but was eventually diagnosed as ccRCC upon pathological examination. In all three cases, our cfDNA-based model predictions were concordant with the final pathological diagnoses. Collectively, these representative cases illustrate the discordance that can occur between imaging-based assessment and final pathology. The concordance between model predictions and pathological diagnoses in these cases suggests that the cfDNA-based model may provide complementary diagnostic information, particularly in radiologically ambiguous cases.

### Exploratory evaluation in an independent oncocytoma cohort

Distinguishing malignant tumors from solid benign lesions, such as AML and oncocytoma, remains a significant clinical challenge due to their overlapping imaging features. We therefore performed a sensitivity analysis restricted to solid benign renal masses to evaluate model performance in this clinically relevant setting. Notably, the model maintained strong discriminatory performance in this subgroup (validation AUC: 0.959; Fig. S11a-c), comparable to that observed in the full benign cohort (validation AUC: 0.946; Fig. 3a-c). These findings suggest that model performance was not primarily driven by the inclusion of more readily distinguishable benign entities and remained stable even after excluding biologically heterogeneous conditions such as renal cysts and nephrolithiasis, which may introduce background cfDNA variation associated with local tissue injury or inflammatory processes.

**Fig. 6:**
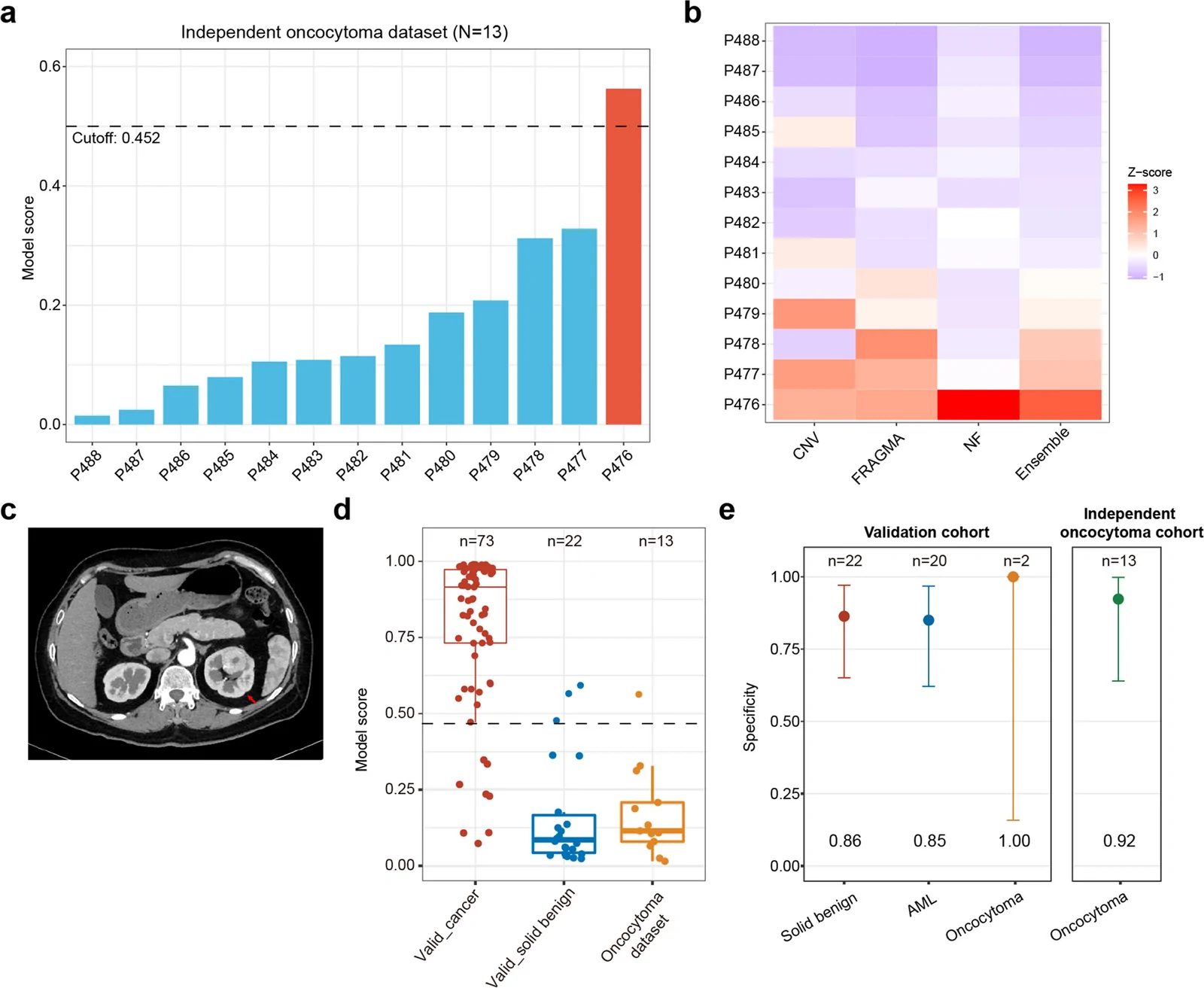
Performance of the model in an independent oncocytoma cohort. **a** Model scores for patients in the independent oncocytoma dataset (N = 13). The dashed line represents the predefined diagnostic cutoff. **b** Heatmap showing Z-score-transformed values of individual model scores for each patient in the independent oncocytoma cohort. Higher Z-scores indicate greater similarity to malignant profiles. **c** Imaging of the patient misclassified as cancer by the model, but pathologically confirmed as oncocytoma. **d** Distribution of model scores in the independent oncocytoma cohort, with validation cancer and solid benign samples shown for comparison. **e** Specificity of the ensemble model in the independent oncocytoma cohort and solid benign subtypes from the validation dataset

To further assess performance in oncocytoma, we analyzed an independent cohort of 13 samples to address their limited representation in the training and validation cohorts. As shown in Fig. 6a, 12 of 13 oncocytoma samples were correctly classified, corresponding to a specificity of 0.92. The single misclassified case showed elevated scores across multiple feature components, suggesting concordant multi-feature signals (Fig. 6b). CT imaging of this case also demonstrated features that were difficult to distinguish from malignant renal tumors based on radiographic assessment alone (Fig. 6c). Despite this challenging case, the overall prediction score distribution of oncocytoma samples more closely resembled that of solid benign lesions than RCC cases in the validation cohort, with consistent performance observed between validation solid benign lesions and the independent oncocytoma cohort (Fig. 6d, e). These results support the robustness of the model in clinically relevant solid renal mass settings, although further validation in larger cohorts is warranted.

## Discussion

In this study, we developed and validated a cfDNA fragmentomics-based machine learning model for noninvasive differentiation of malignant and benign renal masses, addressing a critical unmet need in preoperative clinical management. By integrating multiple cfDNA-derived features, the model achieved high diagnostic accuracy across independent cohorts, tumor burden levels, stages, grades, and pathological subtypes. These results support its potential utility in preoperative risk stratification and clinical decision-making.

Our findings suggest that cfDNA fragmentation patterns capture malignancy-associated biological signals that enable discrimination between malignant and benign renal conditions. Consistent with the notion that global hypomethylation is a hallmark of malignancy [21, 22], cancer patients exhibited lower overall methylation levels compared with benign cases. Functional enrichment analyses further revealed that transcription factors associated with cancer-specific nucleosome footprinting and chromatin architecture were enriched in developmental and reproductive Gene Ontology (GO) terms. This likely reflects intrinsic developmental programming of renal tissue, which originates from the intermediate mesoderm [23, 24]. In malignant cells, developmental regulatory programs involved in kidney development may become aberrantly reactivated or dysregulated, potentially contributing to the observed cancer-associated chromatin patterns. Importantly, these tumor-associated chromatin and epigenetic alterations may be reflected in cfDNA fragmentation patterns, providing a biological basis for distinguishing malignant from benign renal lesions.

Previous cfDNA-based studies have demonstrated the potential of liquid biopsy for renal cancer detection. Although targeted methylation panels and cell-free methylated DNA immunoprecipitation and high-throughput sequencing (cfMeDIP-seq) approaches reported high diagnostic performance [25, 26], they often relied on predefined genomic regions and were primarily designed to distinguish cancer from healthy individuals, a substantially easier task due to the more pronounced molecular differences between groups. More recently, Peng et al. [27] developed a cfDNA fragmentomics assay that included a subset of benign renal conditions and achieved high AUCs of 0.966 and 0.952 in internal and external validation cohorts, respectively. While an important step forward, the diversity and proportion of benign renal masses were limited, leaving uncertainty about performance in truly radiologically ambiguous cases. Similarly, Zhang et al. [28] conducted a multicenter study using motif-based fragmentomic features for early detection of urological tumors, primarily focused on ccRCC. Their model achieved very high AUCs for ccRCC vs non-tumor controls; however, benign renal masses were minimally represented, and the task primarily involved distinguishing cancer from largely healthy or non-renal benign controls. In contrast, our model was specifically designed to address the clinically challenging task of differentiating malignant tumors from radiologically ambiguous benign renal masses, particularly solid lesions such as lipid-poor AMLs and oncocytomas. By integrating complementary fragmentomic features, our model captures broader tumor-associated biological signals not confined to predefined genomic loci, potentially improving robustness and scalability in real-world clinical settings. Clinically, these biological signals enabled accurate classification of diagnostically challenging renal masses. This performance was particularly notable given the low TF observed in most RCC samples in our cohorts, which represents a fundamental limitation for conventional mutation- and copy number-based liquid biopsy approaches.

Notably, most cancer samples exhibited low TF in our study, yet the model maintained robust performance even in this low-shedding context. Performance remained stable after restricting analyses to solid benign renal masses, suggesting that classification was not primarily driven by the inclusion of more readily distinguishable benign entities. One possible explanation is that excluding biologically heterogeneous benign entities may reduce heterogeneity related to non-malignant tissue injury and inflammatory processes, particularly in nephrolithiasis, which is associated with renal injury and inflammation [29, 30]. Furthermore, despite limited representation of oncocytoma in the training and validation cohorts, the model showed consistent performance in an independent oncocytoma cohort, supporting its potential applicability to diagnostically challenging benign renal tumors that frequently overlap with RCC on imaging. The single misclassified oncocytoma case exhibited concordantly elevated scores across multiple feature components, further suggesting that classification decisions were driven by integrated fragmentomic signals rather than isolated feature fluctuations. Together, these findings suggest that cfDNA fragmentomic features capture broader malignancy-associated biological alterations beyond conventional radiographic characteristics. Nevertheless, additional validation in larger multicenter cohorts enriched for rare benign renal tumors will be important to further assess model generalizability and clinical utility.

Clinically, the model demonstrated consistent performance across relevant disease contexts, including early-stage disease characterized by low tumor burden. Nearly half of cancer patients in our cohorts were diagnosed at TNM stage I, which typically presents with small lesion size and low ctDNA abundance, representing a major challenge for existing mutation- or methylation-based cfDNA assays. Nevertheless, our model retained a high sensitivity of at least 0.77 in stage I and 0.90 in stage II disease, with prediction scores increasing progressively from stage I to IV, supporting the association between prediction scores and tumor aggressiveness. From a decision-making perspective, the model could complement size-based clinical assessment, providing biologically informed risk stratification that can refine preoperative management. For small renal masses (≤ 4 cm), where surgical decision-making often requires careful consideration to balance early cancer detection against potential overtreatment, the model detected 84.6% of malignant lesions, suggesting that it may help identify patients who require closer evaluation and timely clinical management, thereby potentially reducing the risk of delayed diagnosis. Conversely, for lesions > 4 cm, in which surgical intervention is more commonly considered in clinical practice, the model correctly identified 85.3% of benign lesions, suggesting a potential role in flagging benign masses before treatment decisions are finalized, which could help avoid unnecessary nephrectomies and preserve renal function when clinically appropriate. By reducing overtreatment, cfDNA fragmentomics has the potential to preserve renal function and improve patients’ QOL, minimizing surgical morbidity, long-term renal impairment, and associated psychosocial burden. Together, our findings indicate that cfDNA fragmentomics not only captures biologically relevant signals across disease stages but also provides decision-making support beyond conventional management paradigms, providing a potential approach to refine current management strategies for renal masses.

Despite these encouraging results, several limitations should be acknowledged. First, although a separate held-out validation cohort was used to assess model robustness, all participants were recruited from a single cancer center. External validation across multiple institutions will be necessary to further establish the generalizability and real-world applicability of our model. Second, certain benign subtypes, particularly oncocytomas, were underrepresented, which may limit statistical power and the precision of performance estimates for these clinically relevant lesions. This is largely attributable to the intrinsic rarity of oncocytoma, which accounts for approximately 3–7% of renal masses in clinical practice [31], making large-scale recruitment inherently challenging. Despite the inclusion of an independent oncocytoma cohort for exploratory evaluation, the overall number of cases remains limited, and larger studies will be required to further validate performance in this subgroup. Third, this study focused on preoperative diagnostic classification and did not include longitudinal follow-up. Consequently, whether cfDNA fragmentomics correlates with recurrence, progression, or survival remains unclear. Fourth, our model was developed and validated in a surgically ascertained cohort of patients with renal masses or other renal lesions, with pathological diagnosis serving as the reference standard, and imaging evaluation in these cohorts relied primarily on contrast-enhanced CT. In routine practice, not all patients undergo MRI or contrast-enhanced ultrasound due to availability, cost, and clinical necessity. As a result, the model’s performance may not fully generalize to incidentally detected renal lesions or to populations evaluated using broader or multimodal imaging approaches. Future studies incorporating unselected, incidentally detected renal lesions and diverse imaging modalities are warranted to further assess model applicability in a real-world renal mass evaluation.

In conclusion, our study demonstrates that cfDNA fragmentomics provides a highly accurate, noninvasive approach for distinguishing malignant from benign renal masses. By capturing tumor-specific chromatin and epigenetic signals, this approach holds promise for improving preoperative risk stratification, reducing unnecessary surgery, and guiding precision management of patients with renal masses.

## Materials and methods

### Participants and samples

This study included a training cohort of 338 participants (173 cancer and 165 benign) and a validation cohort of 150 participants (75 cancer and 75 benign), all of whom had renal masses pathologically confirmed after surgery. Participants were recruited from The First Affiliated Hospital of Army Medical University between April 6, 2023 and August 30, 2024 (training cohort), and between September 1, 2024 and April 14, 2025 (validation cohort), respectively.

Participants were adults (≥ 18 years) who provided written informed consent. For the benign cohort, participants had renal masses or other renal lesions identified by imaging, underwent surgical treatment, and were subsequently confirmed by postoperative pathological examination to have benign renal conditions. For the renal cancer cohort, participants had radiologically suspected renal tumors, underwent surgical treatment, and were postoperatively confirmed to have renal cell carcinoma (RCC).

Participants were withdrawn if the investigator deemed continuation medically inappropriate or if the participant voluntarily requested withdrawal. Exclusion criteria for both cohorts included prior neoadjuvant, adjuvant, or other therapeutic interventions before blood sampling; febrile illness or acute inflammatory disease requiring treatment within 14 days before sampling; oral or intravenous glucocorticoid use within 14 days before sampling; organ transplant recipients or prior non-autologous (allogeneic) bone marrow or stem cell transplantation; Eastern Cooperative Oncology Group (ECOG) performance status > 2; a history of renal cancer or other malignancy within the past five years (for the cancer cohort only); and any other clinically significant condition that could interfere with study compliance or the ability to provide informed consent.

To further support clinical interpretation and evaluate model behavior in underrepresented benign renal tumor subtypes, an additional cohort of 13 patients with pathologically confirmed oncocytoma was enrolled from the Department of Urology, Jiangbei Campus, The First Affiliated Hospital of Army Medical University (approval no. 2022–468-01). These samples were not used for model training or internal validation and were analyzed separately to assess model performance in oncocytoma cases.

All participants underwent blood sampling, and plasma cfDNA was extracted and subjected to low-pass whole-genome sequencing (WGS). The study involving the training and validation cohorts was approved by the Ethics Committee of The First Affiliated Hospital of Army Medical University (approval no. KY2023091). All study procedures were conducted in accordance with international guidelines for good clinical practice. Written informed consent was obtained from all participants before sample collection.

### cfDNA extraction and whole-genome sequencing

Approximately 10 mL of peripheral blood was collected from each participant and processed for cfDNA extraction within 48 h to preserve sample integrity. Plasma was isolated using a two-step centrifugation protocol consisting of an initial spin at 1,600 × g for 10 min at 4 °C, followed by centrifugation of the supernatant at 16,000 × g for 10 min at 4 °C to remove residual cellular debris. cfDNA was extracted using the GENESEEQMag Cell-Free DNA Isolation Kit on the Hamilton Microlab STAR automated workstation (Hamilton, United States) and quantified with the Qubit dsDNA HS Assay Kit (Thermo Fisher Scientific, United States). Purified cfDNA samples were stored at −80 °C until library preparation. Qualified samples were required to have a total cfDNA yield of at least 4 ng and a plasma concentration of ≥ 2 ng/mL.

Sequencing libraries were prepared from 5–10 ng of cfDNA using reagents from the Sample Collection Kit and automated on the Biomek platform (Beckman Coulter, United States). Library concentrations were quantified by quantitative PCR (Vazyme, China). WGS was performed on the DNBSEQ-T7 platform (MGI Tech, China) with 150 bp paired-end reads at an average depth of 5x, ensuring sufficient resolution for fragmentomic profiling. Sequencing was performed across four batches for the training cohort and two batches for the validation cohort, with each batch containing a balanced mixture of cancer and benign samples. The independent oncocytoma cohort was processed as an additional batch. All samples were processed using the same sequencing workflow, reagents, and predefined quality control procedures. Principal component analysis (PCA) was performed across all sequencing batches to assess potential technical variation, and no evident batch-specific clustering was observed (Fig. S12). Samples were included for downstream analysis only if they met all predefined quality control criteria. Specifically, samples were required to exhibit a cfDNA fragment size profile consistent with expected biological characteristics, with a dominant peak within the nucleosomal range (approximately 150–200 bp), a proportion of mappable reads exceeding 98%, a duplication rate below 5%, and a minimum deduplicated sequencing depth of 3x (Additional file 1). Tumor fraction (TF) was inferred through copy number analysis of plasma cfDNA using ichorCNA [32].

### cfDNA fragmentation features

Three cfDNA fragmentomics features, including copy number variation (CNV), fragmentation-based methylation (FRAGMA), and nucleosome footprint (NF), were extracted from low-pass WGS to characterize tumor-specific cfDNA profiles.

#### Copy number variation (CNV)

The whole genome was partitioned into fixed-length bins (2,475 bins of 1 Mb each), and CNVs were inferred using ichorCNA (version 0.3.2). This algorithm applies a Hidden Markov Model (HMM) to read-depth coverage data to identify genomic regions with abnormal copy numbers. For each genomic bin, ichorCNA computes a log2 ratio, defined as the ratio between the observed and expected read counts within that bin. This log2 ratio serves as the key metric for determining copy number alterations: values near zero indicate diploid regions, whereas negative and positive values suggest deletions and amplifications, respectively.

#### Fragmentation-based methylation (FRAGMA)

FRAGMA profiling infers CpG methylation status by analyzing cfDNA fragmentation patterns, providing potential biomarkers for noninvasive prenatal testing, organ transplantation monitoring, and cancer assessment [22]. Specifically, FRAGMA first analyzes all CpG sites located within Alu regions. For these sites, it computes the fragment ratios of eight possible 5’-end cleavage patterns, CGN and NCG (where N represents A, T, C, or G), as well as the fragment count ratio (CGN/NCG), yielding a total of nine features. Subsequently, FRAGMA extends the analysis genome-wide to all CGCG sites, calculating fragment ratios for ten possible 5’-end cleavage patterns involving CGC and NCG (C1), and CGN and GCG (C2), as well as the ratios CGC/NCG and CGN/GCG, generating an additional 12 features. Together, these 21 features comprehensively capture cfDNA fragmentation signatures associated with methylation-dependent nucleosomal protection.

#### Nucleosome footprint (NF)

NF profiling focuses on coverage patterns around transcription factor binding sites, which reflect nucleosome positioning and epigenetic regulation, thereby capturing tumor-associated epigenetic alterations [33]. Specifically, a total of 854 transcription factors were obtained from the Gene Transcription Regulation Database (GTRD) [34]. For each binding site, coverage depth was computed and corrected for GC content to eliminate GC bias. Three key features were then extracted for each transcription factor: (1) core depth, defined as the average coverage within ±30 bp of the binding site; (2) mean depth, defined as the average coverage within ±1 kb of the active transcription factor region; and (3) nucleosome amplitude, representing the oscillatory signal of nucleosome phasing derived from fast Fourier transform (FFT) analysis of the coverage profile. Together, these features quantify cfDNA fragmentation and chromatin accessibility patterns associated with malignant transformation.

### Model development and validation

We employed the H2O AutoML framework [35] to train feature-specific base models using the three cfDNA fragmentomic feature sets and subsequently integrated their predictions into an ensemble for malignancy classification. For each cfDNA feature type, H2O AutoML automatically trained a set of base models on the training cohort, using fivefold cross-validation for internal model selection and hyperparameter fine-tuning to ensure reliable performance estimation. A suite of machine learning algorithms was explored during the H2O AutoML training process, including Distributed Random Forest (DRF), Extremely Randomized Trees (XRT), Generalized Linear Model (GLM), Extreme Gradient Boosting (XGBoost), Gradient Boosting Machine (GBM), and Deep Learning. The Deep Learning algorithm is a fully connected multi-layer feed-forward neural network, trained with stochastic gradient descent using back-propagation. For the random-search hyperparameter tuning, XGBoost, GBM, and Deep Learning algorithms used the default settings described in H2O AutoML documentation. A single GLM model was trained with lambda-search enabled and evaluated on a set of alpha values, returning the best alpha-lambda combination. This is a glmnet-like approach rather than traditional GLM training. Note that DRF/XRT were trained with H2O default settings but not with hyperparameter grid search during AutoML training. All other parameters were left at their H2O defaults. During training, the H2O framework iteratively adjusted key model parameters based on the cross-validation performance until either convergence or a predefined maximum number of iterations was reached.

Base models for each cfDNA feature type were ranked by the area under the receiver operating characteristic curve (AUROC) on their specific leaderboard of cross-validation performance. The top five models with the highest cross-validated AUROC were selected, yielding a total of 15 high-performing base models. To generate the final prediction score, we applied the averaging ensemble strategy, in which the predicted probabilities from each of the top base models were averaged to produce a single integrated prediction. This ensemble approach reduces model-specific variance and enhances generalization and stability across datasets. The final model was locked after training and applied directly to the validation cohort without any retraining or tuning. Thus, the validation cohort served as a fully held-out dataset for out-of-sample performance assessment.

### Feature-level permutation importance test

To evaluate the contribution of each feature set (FRAGMA, CNV, and NF) to the overall classification performance, we performed a permutation-based analysis on the validation set. For each feature set, all corresponding feature columns were independently shuffled across samples to disrupt their association with the outcome while preserving the marginal distribution of each feature. The permuted inputs were then fed into the five base models trained specifically on that feature set. The resulting prediction scores were combined with the original, unpermuted scores from the other two feature sets to generate a new ensemble score, defined as the mean of the five updated predictions and the ten unchanged predictions. This procedure was repeated 100 times for each feature set using independent random permutations. The resulting distributions of permuted AUC values were subsequently compared with the original ensemble AUC to assess the relative contribution of each feature set to overall model performance.

### Immunohistochemistry analysis

Immunohistochemistry (IHC) was performed on formalin-fixed, paraffin-embedded (FFPE) tissue sections using standard protocols. Briefly, 4-µm sections were deparaffinized, rehydrated, and subjected to antigen retrieval, followed by quenching of endogenous peroxidase activity. Slides were then incubated with a panel of primary antibodies at manufacturer-recommended dilutions. The antibodies used in this study included CK (AE1/AE3, ready-to-use, Maixin), CK7 (EP16, ready-to-use, ZSGB-BIO), CA9 (polyclonal, ready-to-use, Maixin), CD10 (SP76, ready-to-use, Ventana), Vimentin (V9, ready-to-use, Ventana), PAX8 (JE80-01, ready-to-use, WELISHPOOL BIO-TECH), EMA (GP1.4, 1:140, ZSGB-BIO), RCC (PN-15, ready-to-use, Maixin), TFE-3 (MRQ-37, ready-to-use, Maixin), Ki-67 (30–9, ready-to-use, Ventana), and GATA3 (EP368, ready-to-use, Maixin). Signal detection was carried out using a biotin-streptavidin-HRP system with diaminobenzidine (DAB) as the chromogen, followed by hematoxylin counterstaining. The Ki-67 proliferation index was determined by calculating the percentage of positively stained nuclei across at least five high-power fields. All IHC slides were independently reviewed by two experienced pathologists.

### Statistical analysis

All statistical analyses were performed using R software (version 4.3.2). Categorical variables were compared using the Chi-square test or Fisher’s exact test, and continuous variables using the Wilcoxon rank-sum test or Kruskal–Wallis test, as appropriate. Trends across ordered groups were evaluated using the Jonckheere-Terpstra test. Diagnostic performance metrics, including true positives (TP), true negatives (TN), false positives (FP), and false negatives (FN), were calculated using the “epiR” package, from which sensitivity and specificity with corresponding 95% confidence intervals (CIs) were derived. Receiver operating characteristic (ROC) curves and area under the curve (AUC) values were generated using the “pROC” package. Logistic regression was used to assess potential effect modification by sex using a model score-by-sex interaction term. Multivariable logistic regression was performed to evaluate the association between the standardized model prediction score and malignancy after adjustment for relevant demographic and clinical covariates, with odds ratios (ORs) reported per 1-standard deviation (SD) increase in the model prediction score. Decision-curve analysis (DCA) was performed to determine the potential clinical usefulness of the models across a range of threshold probabilities, with 95% CIs estimated using bootstrapping. Unless otherwise specified, all statistical tests were two-sided, and a P value < 0.05 was considered statistically significant.

## Supplementary Information

Supplementary Material 1.

Supplementary Material 2.

## Data Availability

The datasets generated and/or analyzed during the current study have been deposited in the Genome Sequence Archive for Human (GSA-Human, https://ngdc.cncb.ac.cn/gsa-human) under accession number HRA018304. Access to the data is managed in accordance with GSA-Human policies for human genomic data and is available upon reasonable request to the corresponding author.
