# Supplementary material for: A cfDNA fragmentomics classifier for noninvasive differentiation of benign and malignant renal masses
Source: Mol Biomed. 2026 Sep 7;7:163. doi: 10.1186/s43556-026-00568-4 (PMC13550314; doi:10.1186/s43556-026-00568-4)
Supplement: Supplementary file 1 [file 43556_2026_568_MOESM1_ESM.docx]

**Supplementary Materials**

**Manuscript title: A cfDNA fragmentomics classifier for noninvasive differentiation of benign and malignant renal masses**

Linfei Li^1†^, Cong Wang^1†^, Song Wang^2†^, Jun Zheng^1^, Jian Fu^1^, Ling Wei^1^, Juan Shen^1^, Yuwei Li^1^, Xinyue Hong^2^, Chunman Wu^2^, Wei Chen^3*^, Wei Gong^4*^, Yongquan Wang^1*^

1 Department of Urology, The First Affiliated Hospital of Army Medical University, Chongqing 400038, China

2 Geneseeq Research Institute, Nanjing Geneseeq Technology Inc., Nanjing 210032, China

3 Department of Urology of Jiangbei Campus, The First Affiliated Hospital of Army Medical University (The 958th Hospital of Chinese People’s Liberation Army), Chongqing 400020, China

4 Department of Biochemistry and Molecular Biology, College of Basic Medical Sciences, Army Medical University, Chongqing 400038, China

†**These authors contributed equally to this work**

***Co-corresponding authors**

**Yongquan Wang** ([wyq@tmmu.edu.cn](mailto:wyq@tmmu.edu.cn)); **Wei Gong** ([gongwei@tmmu.edu.cn](mailto:gongwei@tmmu.edu.cn)); **Wei Chen** ([doctorcw@tmmu.edu.cn](mailto:doctorcw@tmmu.edu.cn))

## **Supplementary Tables**

| **Table S1. Sensitivity escalation analysis** | | | | |
| --- | --- | --- | --- | --- |
| Target Sensitivity | Dataset | Cutoff | Sensitivity (95% CI) | Specificity (95% CI) |
| 90% | Train | 0.452 | 0.901 (0.856–0.942) | 0.900 (0.852–0.945) |
| 90% | Valid | 0.452 | 0.892 (0.817–0.957) | 0.872 (0.791–0.945) |
| 95% | Train | 0.189 | 0.953 (0.921–0.982) | 0.662 (0.586–0.735) |
| 95% | Valid | 0.189 | 0.959 (0.910–1.000) | 0.661 (0.547–0.768) |
| 98% | Train | 0.138 | 0.983 (0.961–1.000) | 0.562 (0.484–0.635) |
| 98% | Valid | 0.138 | 0.959 (0.910–1.000) | 0.618 (0.500–0.726) |

| **Table S2. Clinical characteristics of misclassified tumors at various sensitivity cutoffs.** | | | |
| --- | --- | --- | --- |
| **Characteristics** | **Sens_90 (N=25)** | **Sens_95 (N=11)** | **Sens_98 (N=6)** |
|  |  |  |  |
| **Age** | 59.0 [30.0, 78.0] | 61.0 [30.0, 75.0] | 59.5 [32.0, 75.0] |
| **Sex** |  |  |  |
| Female | 8 (32.0%) | 3 (27.3%) | 2 (33.3%) |
| Male | 17 (68.0%) | 8 (72.7%) | 4 (66.7%) |
| **Hypertension status** |  |  |  |
| Normotensive | 18 (72.0%) | 7 (63.6%) | 4 (66.7%) |
| Grade 1 | 3 (12.0%) | 1 (9.1%) | 1 (16.7%) |
| Grade 2 | 2 (8.0%) | 1 (9.1%) | 1 (16.7%) |
| Grade 3 | 2 (8.0%) | 2 (18.2%) | 0 (0%) |
| **Smoking history** |  |  |  |
| Never | 14 (56.0%) | 3 (27.3%) | 2 (33.3%) |
| Current/former | 11 (44.0%) | 8 (72.7%) | 4 (66.7%) |
| **TNM stage** |  |  |  |
| I | 22 (88.0%) | 9 (81.8%) | 6 (100%) |
| II | 2 (8.0%) | 1 (9.1%) | 0 (0%) |
| III | 1 (4.0%) | 1 (9.1%) | 0 (0%) |
| IV | 0 (0%) | 0 (0%) | 0 (0%) |
| **WHO/ISUP grade** |  |  |  |
| I | 5 (20.0%) | 3 (27.3%) | 1 (16.7%) |
| II | 13 (52.0%) | 4 (36.4%) | 4 (66.7%) |
| III | 1 (4.0%) | 0 (0%) | 0 (0%) |
| IV | 0 (0%) | 0 (0%) | 0 (0%) |
| Unknown | 3 (12.0%) | 2 (18.2%) | 0 (0%) |
| Not applicable | 3 (12.0%) | 2 (18.2%) | 1 (16.7%) |
| **Radiological size** |  |  |  |
| Size ≤ 4 cm | 16 (64.0%) | 7 (63.6%) | 4 (66.7%) |
| 4 cm < Size ≤10 cm | 8 (32.0%) | 3 (27.3%) | 2 (33.3%) |
| Size >10 cm | 1 (4.0%) | 1 (9.1%) | 0 (0%) |
| **Pathological type** |  |  |  |
| ccRCC | 18 (72.0%) | 6 (54.5%) | 4 (66.7%) |
| chRCC | 2 (8.0%) | 1 (9.1%) | 1 (16.7%) |
| pRCC | 4 (16.0%) | 3 (27.3%) | 1 (16.7%) |
| Others | 1 (4.0%) | 1 (9.1%) | 0 (0%) |
| Abbreviations: TNM, tumor, node, metastasis; WHO, World Health Organization; ISUP, International Society of Urological Pathology; ccRCC, clear cell renal cell carcinoma; chRCC, chromophobe renal cell carcinoma; pRCC, papillary renal cell carcinoma; Sens, sensitivity | | | |

| **Table S3.** **Scenario analysis of PPV and NPV for the model under varying disease prevalence** | | | | | |
| --- | --- | --- | --- | --- | --- |
| Dataset | Prevalence | Sensitivity (95% CI) | Specificity (95% CI) | PPV (95% CI) | NPV (95% CI) |
| Training | 1% | 0.901 (0.856–0.942) | 0.900 (0.852–0.945) | 0.083 (0.078-0.089) | 0.999 (0.999-0.999) |
|  | 5% |  |  | 0.322 (0.306-0.337) | 0.994 (0.994-0.995) |
|  | 10% |  |  | 0.500 (0.482-0.518) | 0.988 (0.987-0.989) |
|  | 20% |  |  | 0.692 (0.677-0.708) | 0.973 (0.971-0.975) |
|  | 30% |  |  | 0.794 (0.782-0.806) | 0.955 (0.952-0.958) |
|  | 50% |  |  | 0.900 (0.893-0.906) | 0.901 (0.894-0.907) |
|  | 70% |  |  | 0.955 (0.951-0.958) | 0.795 (0.783-0.807) |
|  | 80% |  |  | 0.973 (0.971-0.975) | 0.694 (0.678-0.709) |
| Validation | 1% | 0.892 (0.817–0.957) | 0.872 (0.791–0.945) | 0.066 (0.059-0.074) | 0.999 (0.999-0.999) |
|  | 5% |  |  | 0.270 (0.247-0.294) | 0.993 (0.993-0.994) |
|  | 10% |  |  | 0.438 (0.409-0.468) | 0.986 (0.985-0.988) |
|  | 20% |  |  | 0.637 (0.609-0.664) | 0.970 (0.966-0.973) |
|  | 30% |  |  | 0.751 (0.728-0.772) | 0.949 (0.943-0.954) |
|  | 50% |  |  | 0.875 (0.862-0.888) | 0.888 (0.876-0.900) |
|  | 70% |  |  | 0.942 (0.936-0.949) | 0.773 (0.752-0.794) |
|  | 80% |  |  | 0.966 (0.961-0.969) | 0.666 (0.639-0.692) |
| Abbreviations: PPV, positive predictive value; NPV, negative predictive value; CI, confidence interval | | | | | |

## **Supplementary Figures**

### **Figure S1. Relative importance of features**

(**a**) Comparison of model performance across training and validation cohorts. Bars represent the “full model” integrating all features from 15 base models (dark blue), “Drop-one” models where one feature group was excluded (orange), and “Single” models utilizing only one feature group (light blue). (**b**) Feature importance evaluated by permutation testing on the validation cohort. The dashed line indicates the baseline area under the curve (AUC; 0.946) of the full model. Each boxplot represents the distribution of permuted AUC values after 100 random shuffles of the indicated feature group. CNV, copy number variation; FRAGMA, fragmentation-based methylation; NF, nucleosome footprint.

### **Figure S2. Decision curve analysis of predictive models**

Clinical utility was evaluated by plotting standardized net benefit across a range of risk thresholds in the training (**a**) and validation (**b**) cohorts. The performance of the ensemble model was compared with that of individual feature-based models. CNV, copy number variation; FRAGMA, fragmentation-based methylation; NF, nucleosome footprint.

### **Figure S3. Assessment of potential demographic bias**

(**a, b**) Distribution of model scores stratified by sex in the training (a) and validation (b) cohorts. (**c**) Predicted probability of malignancy as a function of model score, stratified by sex. (**d**) Forest plot of multivariable logistic regression analyses evaluating the independent predictive value of the model score. Odds ratios (OR) and 95% confidence intervals (CI) were adjusted for age, sex, and the interaction between model score and sex. (**e**) Receiver operating characteristic curves of the predictive model stratified by sex in the training and validation cohorts. (**f**) Sensitivity and specificity of the model across sex-stratified subgroups.

### **Figure S4. Multivariable logistic regression model of factors associated with malignant renal mass**

Forest plots show adjusted odds ratios (ORs) and 95% confidence intervals from multivariable logistic regression analyses in the training and validation cohorts. The primary model included model score, age, sex, BMI, hypertension, smoking history, and radiological tumor size. The sensitivity model additionally included triglycerides and total cholesterol. The model score was standardized within each cohort, with ORs reported per 1-SD increase.

### **Figure S5. Tumor fraction estimation of cancer samples**

(**a**) Density plots showing the distribution of tumor fraction (TF) in the training and validation cohorts. Vertical dashed lines indicate the cohort-specific median TF values, with medians and interquartile ranges (IQR) annotated. (**b**) Stacked bar plot showing the proportion of cancer samples stratified by the 3% TF threshold.

### **Figure S6. ROC curves for the predictive model in different subgroups**

Receiver operating characteristic (ROC) curves are shown stratified by TNM stage (**a**), WHO/ISUP grade (**b**), pathological type (**c**), benign type (**d**), and radiological size (**e**).

### **Figure S7. Model prediction scores across datasets in different subgroups**

Box plots showing the distribution of model prediction scores stratified by TNM stage (**a**), WHO/ISUP grade (**b**), pathological type (**c**), and benign type (**d**) across the training and validation datasets.

### **Figure S8. Distribution of model prediction scores between benign and cancer samples stratified by clinical features**

Box plots showing the distribution of model scores for benign and cancer samples, stratified by various baseline characteristics in the training and validation cohorts, including hypertension status (**a**), smoking history (**b**), body mass index (BMI) (**c**), triglyceride levels (**d**), and total cholesterol levels (**e**).

### **Figure S9. Model prediction stratified by radiological size threshold and the predefined cutoff corresponding to 90% sensitivity across cohorts**

### **Figure S10. Subgroup analysis stratified by radiological size at alternative cutoff values**

(**a, b**) Subgroup analysis of sensitivity (red) and specificity (blue) across different tumor size categories across datasets using two cutoff values: 0.189, which corresponds to 95% sensitivity in the training cohort (a), and 0.138, which corresponds to 98% sensitivity in the training cohort (b). (**c, d**) Scatter plots and confusion matrices detailing model performance at the two cutoffs. The top panels show the distribution of model scores against tumor size for benign (blue) and cancer (red) cases in both training and validation sets, with horizontal dashed lines indicating the respective cutoffs. The lower panels provide confusion matrices stratified by tumor size, reporting absolute counts, sensitivity, and specificity with associated 95% confidence intervals.

### **Figure S11. Diagnostic performance of the model in distinguishing malignant from solid benign renal masses**

(**a**) Receiver operating characteristic (ROC) curves of the predictive model for differentiating malignant tumors from solid benign renal masses (angiomyolipoma and oncocytoma). (**b, c**) ROC curves comparing the ensemble model with single-feature models in the training (b) and validation (c) cohorts, restricted to malignant and solid benign renal masses.

### **Figure S12. Assessment of potential batch effects in cfDNA sequencing data**

Principal component analysis (PCA) was performed across sequencing batches from the training, validation, and independent oncocytoma cohorts to evaluate potential batch effects. The training cohort was processed across four sequencing batches, the validation cohort across two batches, and the oncocytoma cohort as an additional batch. Each point represents an individual sample, colored by sequencing batch.
